# Distortion product otoacoustic emission (DPOAE) reveals hearing loss up to 16 kHz in pediatric chemotherapy patients

**DOI:** 10.1038/s41598-026-47642-z

**Published:** 2026-04-18

**Authors:** Dietmar J. Hecker, Marc K. H. Remke, Maximilian Linxweiler, Rhoikos Furtwängler, Yeliz Akrasu, Dominik Schöndorf, Norbert Graf, Dorothée Krieter, Nadine Oberkircher, Bernhard Schick, Alessandro Bozzato, Arne Simon

**Affiliations:** 1 Department of Otorhinolaryngology, Head and Neck Surgery, Saarland University Hospital, 66421 Homburg, Germany; 2 Department of Pediatrics Hematology/Oncology, Saarland University Hospital, 66421 Homburg, Germany; 3 Divicsion of Pediatric Hematology and Oncology, Inselspital, 3015 Bern, Switzerland; 4 Department of Otorhinolaryngology, Hospital Winsen, 21423 Winsen (Luhe), Germany; 5 Saarland University Medical Center, Kinder- und Jugendpsychiatrie und des Childhoodhaus Saarland, 66421 Homburg, Germany; 6 Department of Otorhinolaryngology, Faculty of Medicine, Saarland University, 66421 Homburg, Germany

**Keywords:** Sensory hearing loss, Normal hearing, Otoacoustic emissions, Outer hair cells, Ototoxicity, Cisplatin, Vincristine, Carboplatin, Pure tone audiometry, Cancer, Medical research, Oncology

## Abstract

In pediatric cancer patients, platinum-induced sensory hearing loss (SHL) manifests in bilateral, symmetrical loss of outer hair cells and starts at a frequency range up to 10 kHz. Hearing loss has a significant impact on education, social integration and personality development in childhood cancer survivors. Early reliable detection of hearing loss may prompt attending oncologists to change chemotherapy if a less ototoxic therapeutic alternative is available. Pediatric cancer patients (2–19 years) receiving cisplatin-, carboplatin- or vincristine-containing regimens were eligible. Ultra-high frequency pure tone audiometry (PTA) and ultra-high frequency DPOAE measurements (up to 16 kHz) were compared. A total of 153 examinations were performed in 83 consecutive patients. While only 60 PTAs yielded reliable results, 153 DPOAE examinations up to 16 kHz were informative. Significant findings were observed between 10 and 16 kHz in both PTA and DPOAE assessments. In the cisplatin group, we found a significant reduction in DPOAE levels from 13 to 16 kHz, as well as a significant increase in DPOAE levels at 2.5 kHz and 3 kHz. Treatment with VCR and carboplatin did not result in substantial SHL. Hearing measurements up to 16 kHz can reveal an early ototoxic effect. In pediatric cancer patients, DPOAE measurement (up to 16 kHz) is more feasible and reliable (compared to PTA) and can detect SHL in ultra-high frequencies (10–16 kHz) at an earlier time point.

## Introduction

With the advent of multimodality and support care, five-year survival in pediatric cancer patients exceeds 80% in most high-income countries^1^. Long term sequelae of chemotherapy, such as ototoxicity, are increasingly becoming a focus in pediatric oncology^2^.

During treatment, pediatric cancer patients may receive more than one ototoxic drug^3^ suspected to cause hearing deficits. The most prominent are platin derivatives but these also include aminoglycoside antibiotics or vancomycin and loop diuretics (e.g. furosemide). Drug toxicity is fostered by cranial radiation with significant exposure to the inner ear. By contrast, ototoxicity is not a common long‑term sequela of vincristine or vinblastine^4,5^. The impact of hearing loss on the education, social integration and personality development of childhood cancer survivors must be addressed, especially since children under five years of age have a significantly higher risk of hearing loss compared to adolescents^6^. Detecting hearing loss is essential, as it may prompt oncologists to change chemotherapy if a less ototoxic therapeutic alternative with the same efficacy is available^7^.

Cisplatin- and carboplatin-induced ototoxicity damages several key structures within the cochlea. The outer hair cells are among the most vulnerable targets, with damage beginning in the high-frequency region of the cochlea^8,9^, where cisplatin triggers oxidative stress, mitochondrial dysfunction, and apoptosis, leading to early high‑frequency hearing loss. The stria vascularis is another major site of injury, as cisplatin accumulates in this tissue and disrupts ion transport processes essential for maintaining the endocochlear potential. This impairment compromises cochlear amplification and overall sensory transduction. In addition, spiral ganglion neurons exhibit susceptibility to cisplatin‑induced inflammation and cell-death pathways, contributing to reduced neural synchrony and poorer speech perception. Together, these effects on the cochlear sensory, metabolic, and neural components form the basis of cisplatin-related auditory dysfunction^10^.

Severe hearing loss is more frequently observed in young children (infants and toddlers), particularly in those receiving cisplatin for brain tumors (e.g. medulloblastoma maintenance treatment), neuroblastoma or hepatoblastoma^11^. Young children are especially vulnerable to cisplatin-induced ototoxicity because the auditory system is still developing, making cochlear structures more susceptible to toxic injury. In addition, immature antioxidant and detoxification pathways result in reduced capacity to neutralize cisplatin-induced oxidative stress, and cisplatin tends to accumulate in the cochlea over time, leading to progressive and irreversible damage^2,12,13^.

Unfortunately, infants and toddlers are not capable of cooperating sufficiently for threshold audiometry^14^. The most valid assessment tool for detecting hearing loss in chemotherapy is ultra-high frequency pure tone audiometry^15,16^. However, this assessment is usually not suitable for children younger than 8 years of age. For preschool children, behavioral audiometry can be used up to 6 kHz. Electrophysiological testing (auditory brainstem responses (ABRs)) and otoacoustic emissions (OAEs) may be useful in younger children or in older children who are non-compliant^17^. However, since ABR testing is susceptible to myogenic artefacts, young or non-cooperative children need to be sedated when this measurement is performed, and typical commercial ABR devices can only estimate hearing thresholds objectively in the frequency range of 4 to 8 kHz^18^. Furthermore, although OAEs are easier to perform than ABRs and may be more sensitive for detecting moderate deafness than behavioral audiometry, interpretation may be difficult, especially in the presence of concomitant middle ear effusion.

OAEs are low-level acoustic signals generated by active sound amplification in cochlear outer hair cells (OHCs) and transmitted into the external auditory canal, where they can be detected by a sensitive microphone^19,20^. To date, two types of OAEs have been recorded in clinical practice: transient evoked OAEs (TEOAEs), and distortion product OAEs (DPOAEs)^20^. TEOAEs are triggered by short click stimuli. Although the click stimuli spans a wide frequency band, TEOAE responses measured in clinical practice are band limited to 1–4 kHz in normal‑hearing subjects^21^. DPOAEs are evoked by two simultaneous pure-tone stimuli. Due to the cochlea’s nonlinear amplification mechanism many intermodulation distortion products are produced. Despite this high number of emitted distortion products, current clinical DPOAE devices only make use of the emitted signal at the frequency component 2*f*_1__−_ *f*_2_ as a diagnostic parameter. Modulation of the stimulus frequencies (*f*_1_ and *f*_2_) between 1 and 8 kHz allows screening of the cochlea for functional integrity along the basilar membrane. The recorded acoustic data are a superposition of the applied pure-tone stimuli and the emitted low-level DPOAE signal 2*f*_1_ − *f*_2_ at about 5–15 dB*/*SPL^19,20,22^.

Taking into consideration that ototoxic drugs (such as platinum-containing chemotherapy) and chronic noise exposure cause high-frequency OHC damage above 8 kHz, a suitable and feasible objective audiometric test in this range would be of outstanding clinical value^8,23^. So far, there have been few approaches to recording high-frequency DPOAEs. Due to their poor signal-to-noise ratio, meatal nodes from standing waves and calibration issues, high-frequency DPOAEs > 8 kHz have hardly been addressed in experimental and clinical audiology so far. For instance, Dreisbach et al*.*^24–26^ and Hecker et al.^27^ have shown that high-frequency DPOAE measurements are reproducible up to 16 kHz or 18 kHz. That this is not a matter of course is shown by Jedrzejczak et al.^28^.

Previous studies concerning hearing loss in pediatric cancer patients receiving cisplatin have reported long-term effects after cessation of chemotherapy or at latest follow up^29^. The primary objectives of this prospective study were 1) to use high-frequency DPOAEs as a new and objective strategy for the detection of chemotherapy-induced ototoxicity in pediatric cancer patients instead of pure tone audiometry. 2) to evaluate whether a signal of reduced OHC function (measured using DPOAEs) can be used as an early, clinically relevant finding in patients with subsequent cisplatin-induced hearing loss.

## Materials and methods

### Ethical standards

The study was performed in accordance with the ethical guidelines of Declaration of Helsinki. Informed consent was obtained from all participants prior to the study. The local physicians’ ethics review board (local ethics board index numbers 40/11 effective 03/21/2011) has obtained ethical approval. In this regard, the experimental protocol was approved by the Ethics Committee of the Saarland Medical Association. Informed consent to perform DPOAE measurements in addition to conventional audiometry was obtained from the children’s parents/or their legal guardian(s) and directly from adolescent patients. At least one parent/legal guardian was present during the examination.

### Subjects

We included all pediatric cancer patients (2–19 years, classified into three age groups: 2–5 years, 6–10 years, and 11 years and older) receiving chemotherapy with cisplatin, carboplatin or a vincristine-containing regimen from 2011 to 2019.We excluded patients with genetically determined preexisting hearing loss (e.g., trisomy 21).

### Audiology measurements

Audiological assessments using the MA 55 audiometer (Maico) with circumaural headphones HDA200, (Sennheiser) included measurements of pure-tone audiometry (PTA) at frequencies of 1, 1.5, 2, 4, 6, 8, 10, 12.5, 14 and 16 kHz, depending on the age and development of the child. For children under 4 years, conditioned play audiometry or visual reinforcement audiometry was used, up to 4 kHz. Otoscopy was performed by a consultant. When otoscopy and/or tympanometry revealed conductive middle ear pathology, the examination was postponed.

To classify hearing loss, we used the International Society of Pediatric Oncology (SIOP) Ototoxicity Scale. In some cases (n = 7), SIOP classification was possible only up to 4 kHz or 6 kHz because the children were too small to provide valid information up to at 8 kHz. In these cases, the hearing loss of the undeterminable sound frequency was estimated to be so high that the SIOP grade was increased one level. In 13 cases, no PTA was possible because the children were not cooperative. All patients in the pretreatment group had to have detectable TEOAE emissions and were not allowed to wear hearing aids. DPOAE measurements were conducted using stimulus levels of L1 = 65 and L2 = 55 dB (A) across f2 ranges of 2–8 kHz and 10–16 kHz in 0.5‑kHz increments, with an f2/f1 ratio of 1.2. All calculated variables represent the mean value of the right and left ears for both hearing thresholds and DPOAE values. Unlike pure‑tone audiometry, which could not be completed in all children, DPOAE testing was feasible in all participants. All examinations were performed without sedation.

### Treatment

The cumulative dose per single cisplatin/carboplatin therapy ranged from 60/400 mg/m^2^ to 160/600 mg/m^2^; the total cumulative dose received over the complete therapy for each drug ranged from 120/800 mg/m^2^ to 320/1.1200 mg/m^2^.

### Statistics

SPSS version 29 (IBM, Ehningen, Germany) was used for statistical analysis using a significance level of 5%. To test the significance of thresholds, normality was assessed using the Kolmogorov–Smirnov test, and homogeneity of variance was assessed using the Levene test. PTA thresholds and DPOAE levels were analyzed using one‑way or two‑way ANOVA when assumptions of normality and homogeneity of variance were met. In cases of variance heterogeneity, Brown-Forsythe‑corrected ANOVA was applied. When data violated normality assumptions, group differences were assessed using the non‑parametric Kruskal–Wallis test (KWT). Although Bonferroni correction is commonly used to account for multiple testing, it requires homogeneity of variances; because this assumption was violated, pairwise comparisons were performed using the Games-Howell procedure. Since hearing assessments were not available at all time points for the same patients and the composition of the sample differed between assessments, the three measurement occasions (pre‑treatment, first treatment, second treatment) were analyzed as independent groups, rather than as a classical repeated‑measures design. A multivariate analysis of variance (MANOVA) was applied to test for overall multivariate effects of the independent variables on the set of correlated outcome measures. Since Box’s M test was significant, indicating heterogeneity of covariance matrices, Pillai’s Trace was used as the primary multivariate statistic due to its robustness. Pearson correlations were used for associations between metric variables, whereas Spearman correlations were applied when at least one variable was ordinal or non-normally distributed. Continuous variables are presented as mean and standard error.

Concerning the sample size, we knew from our previous data that the prevalence of non-interpretable results in the DPOAE group (comprising all pediatric age groups) would be 2.5% (data on file), and the corresponding failure rate of conventional audiometry would be 20%. As a result, we would need 144 corresponding examinations to confirm a significant difference between these two methods.

## Results

### Included patients

During the observation periode, 83 patients (37 females and 46 males, aged 2–19 years, median 11.3, IQR: 6.7 to 14.3 years) were included in the study. Because pure tone audiometry was not feasible for all children, analyses of hearing function were primarily based on DPOAE measurements, which were available for all participants. In cases where the hearing threshold could not be established, the field was left empty. No values were substituted, corrected, or otherwise imputed. Pretreatment measurements were unavailable for many children, as the urgency to begin therapy in severe clinical situations outweighed the possibility of baseline testing.

### Hearing testing and thresholds

**Fig. 1:**
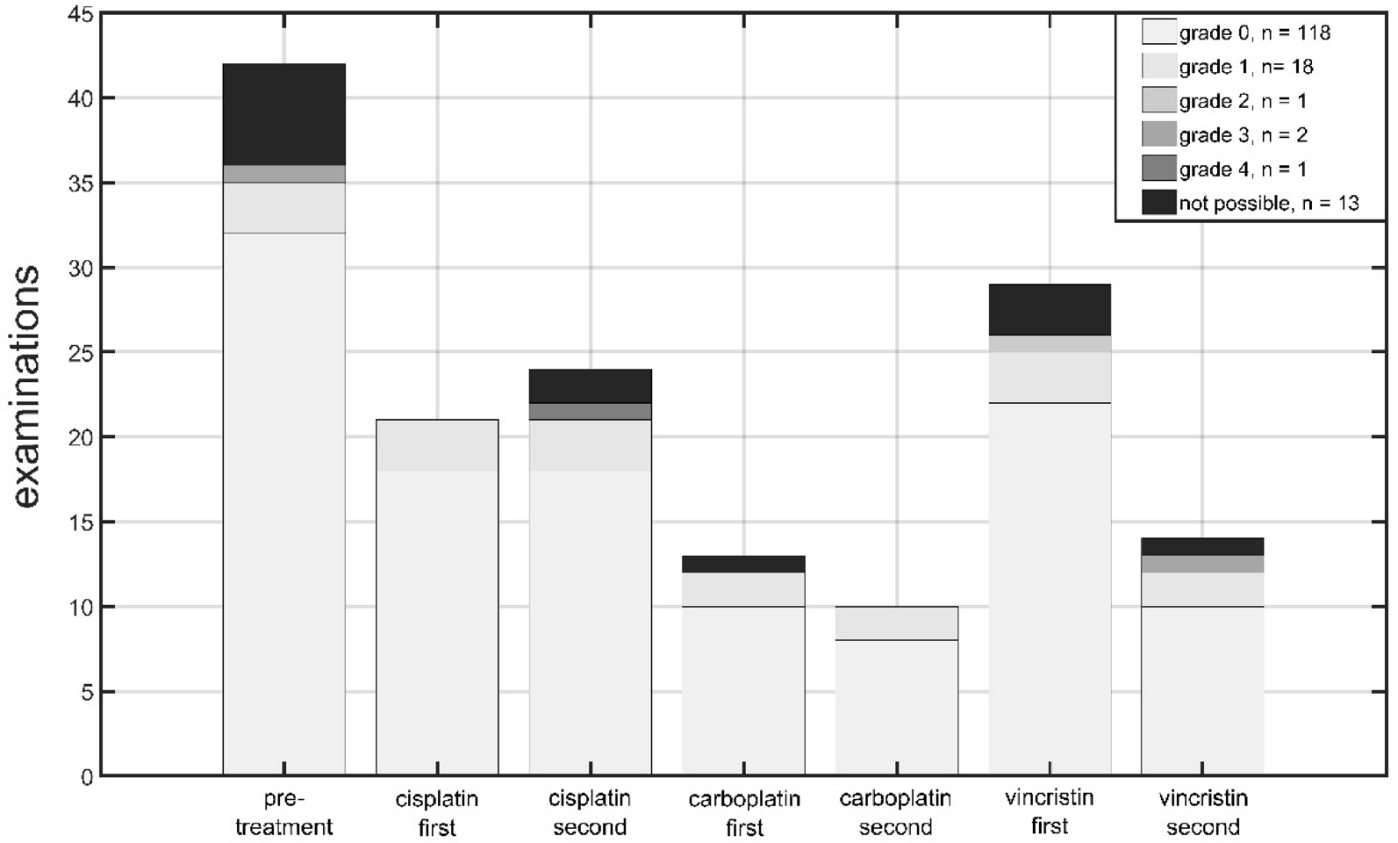
Hearing loss classification from grade 0 to 4 (based on SIOP) colored in gray scale depending on treatment (pretreatment, cisplatin, carboplatin, vincristine) and status of chemotherapy (first, second).

Based on the SIOP grading, Fig. 1 (displaying the results of 153 examinations) shows the detected hearing loss as a stacked bar graph. Each bar represents a treatment, and the slices of each bar show the grades of hearing loss, colored in greyscale. The vast majority (77%) had no hearing loss (grade 0), followed by a grade 1 hearing loss in 12% of patients. In 8% of the whole patient population, a hearing test was not possible due to insufficient cooperation.

To analyze mean PTA, we averaged PTA threshold from 1 to 8 kHz and from 10 to 16 kHz. In total, the results of 140 examinations (153–13 dropouts) are displayed in Table 1, with the data further organized by age groups. Grouped values are presented as mean ± standard error.

**Table 1:** Grouped results are expressed as mean with parentheses specifying the number of observed cases in relation to the total number of eligible patients (first value = measured patients; second value = eligible patients) and standard error.

Group	Children 2 to 5 years (21f./17 m)				Children 6 to 10 years (12f./31 m)				Children up to 11 years (36f./36 m)			
	1 to 8 kHz [dB/HL]		10 to 16 kHz [dB/HL]		1 to 8 kHz [dB/HL]		10 to 16 kHz [dB/HL]		1 to 8 kHz [dB/HL]		10 to 16 kHz [dB/HL]	
	Mean	SE	Mean	SE	Mean	SE	Mean	SE	Mean	SE	Mean	SE
Pre-treatment (22f./20 m)	12.5 (8/12)	3.1	9.1 (4/12)	3.7	4.3 (6/6)	1.3	6.0 (6/6)	2.0	5.5 (22/24)	0.6	7.4 (22/24)	1.4
Cisplatin first (12f./9 m)	17.3 (3/3)	7.6	42.5 (2/3)	42.4	6.9 (7/7)	1.3	17.8 (7/7)	4.2	6.1 (11/11)	0.9	12.8 (11/11)	3.4
Cisplatin second (12f./12 m)	12.7 (3/4)	8.6	12.5 (3/4)	8.7	3.8 (10/10)	0.5	9.6 (10/10)	2.2	8.8 (9/10)	2.3	23.9 (9/10)	7.3
Carboplatin first (3f./10 m)	7.7 (4/4)	1.3	17.6 (4/4)	7.1	5.2 (3/3)	1.1	3.4 (2/3)	0.5	5.8 (5/6)	1.6	10.9 (5/6)	4.0
Carboplatin second (4f./6 m)	9.5 (3/3)	2.4	13.8 (3/3)	9.5	9.2 (4/4)	0.7	16.5 (4/4)	4.7	3.4 (3/3)	0.9	2.8 (3/3)	1.1
VCR frist (11f./18 m)	8.6 (6/7)	1.0	7.3 (4/7)	1.6	3.8 (7/8)	1.2	8.1 (6/8)	3.5	6.5 (14/14)	0.7	9.5 (13/14)	1.8
VCR second (5f./9 m)	9.8 (5/5)	3.0	11.0 (4/5)	2.6	3.2 (5/5)	0.9	10.3 (5/5)	2.5	2.9 (3/4)	1.5	7.4 (3/4)	4.2
*p* value (^c^ Brown-Forsythe correction)	0.805*		0.787*		0.070		**0.044**		0.205*		0.034*	

All calculated thresholds are expressed in dB/HL. Significant *p*-values are presented in bold with an asterisks, and ^c^ indicates values corrected using the Brown-Forsythe procedure.

Table 1 summarizes mean hearing thresholds (± SE) for the frequency ranges 1 to 8 kHz and 10 to 16 kHz across the three age groups and all treatment conditions. In the conventional frequency range (1–8 kHz), thresholds remained relatively stable across treatments and age groups, with no statistically significant differences (one-way ANOVA) after Brown-Forsythe correction. By contrast, high‑frequency thresholds (10–16 kHz) showed greater variability, particularly after cisplatin administration, when elevated mean thresholds were observed across all age groups. Significant differences were detected in the 6–10 year group (*p* ≤ 0.044) and in the combined cohort up to 11 years (*p* ≤ 0.034), indicating a more pronounced high‑frequency susceptibility. No significant differences were found in the youngest age group. Although the ANOVA showed a significant overall effect, neither the Bonferroni—corrected nor the Games-Howell post hoc comparisons revealed significant pairwise differences.

Due to the unequal variances, the two-way ANOVA (age group × treatment) could not be interpreted reliably. A multivariate analysis of variance (MANOVA), including the two hearing measures (1–8 kHz and 10–16 kHz) across the treatments and groups, was not conducted. The sample sizes within several medication groups were too small to meet the assumptions required for a reliable MANOVA.

In contrast to the age-group-stratified analyses shown previously, the table below displays the pooled PTA results without age-group differentiation. It provides mean and median thresholds for 1 to 8 kHz and 10 to 16 kHz, together with standard deviations, confidence intervals, and Brown-Forsythe-adjusted p-values for the respective treatment conditions. A significant difference between treatment conditions was observed for PTA 10 to 16 kHz (*p* ≤ 0.046, Brown-Forsythe corrected), whereas no significant differences were found for PTA 1 to 8 kHz (*p* ≤ 0.936). Although the ANOVA indicated a significant overall effect, neither the Bonferroni-corrected nor the Games-Howell post hoc comparisons revealed significant pairwise differences.

A MANOVA did not reveal any significant multivariate effects of therapy or gender on the combined hearing measures. The univariate effects of therapy on high‑frequency hearing loss are described in the ANOVA section (see Table 2). In addition, no significant main effect of gender was observed for either hearing variable.

**Table 2:** Hearing threshold from the PTA with mean, median, standard deviation and 95% confidence interval levels from 1 to 8 kHz and 10 to 16 kHz are listed.

	**PTA [dB/HL] 1 to 8 kHz**					**PTA [dB/HL] 10 to 16 kHz**				
				**Confidence interval**					**Confidence interval**	
	**Mean**	**Median**	**SD**	**Lower**	**Upper**	**Mean**	**Median**	**SD**	**Lower**	**Upper**
pretreatment	5.8	5.4	3.2	4.6	6.9	7.3	6.8	6.2	5.1	9.6
Cis first*	7.5	6.8	6.2	4.6	10.4	17.5	10.9	19.4	8.4	26.6
Cis sec*	5.9	5.4	5.2	3.6	8.3	15.1	8.5	16.8	7.6	22.8
Carbo first	6.1	5.6	3	4.1	8.2	12	10	11	4.6	19.4
Carbo sec	7.6	7.8	3.6	5	10.1	11.6	7	11.3	3.5	19.7
VCR first	6.2	5.8	2.8	5	7.4	8.8	7.9	6.5	6	11.5
VCR sec	4.6	3.8	4.2	2	7.2	9.8	9.8	5.6	6.2	13.4
*p* value (^c^ Brown-Forsythe correction)	0.936					**0.046**				

The rows represent the therapy status. All calculated values expressed in dB/HL. Significant *p*-values are presented in bold with an asterisks, and ^c^ indicates values corrected using the Brown-Forsythe procedure.

### Distortion product otoacoustic emissions (DPOAEs)

**Fig. 2:**
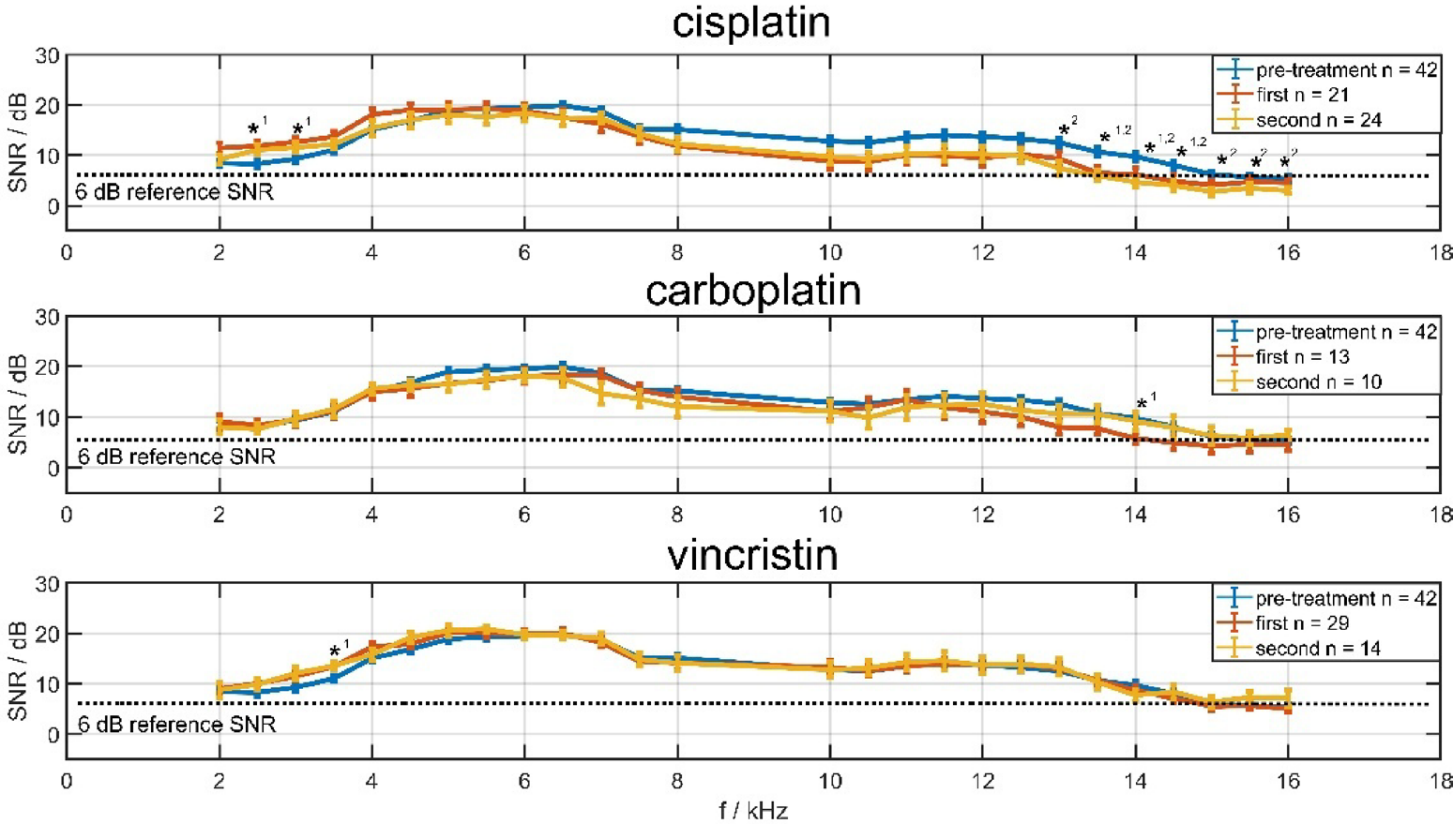
SNR of DPOAE levels for f2 frequencies from 2 to 8 kHz and 10 to 16 kHz (across 0.5 kHz bins) for both ears of all patients depending on treatment (cisplatin, carboplatin, vincristine) and status (pre-treatment, first, second) with mean values and standard errors. The blue line represents hearing measurements of the pretreatment group, the red line represents measurements after the first treatment, and the yellow line represents measurements after the second treatment. Significant differences (based on Kruskal–Wallis testing with Bonferroni correction) from the pre-treatment group are marked with an asterisks and indexed (1-first, 2-s) at some frequencies.

In contrast to pure-tone hearing detection, DPOAE measurements could be performed in both ears of all 153 examinations. Figure 2 shows the average signal-to-noise ratio (SNR) for the DPOAE signal 2f_1_-f_2_ across treatments and therapies. Mean values and standard errors are shown in f_2_ frequencies ranging from 2 to 8 kHz and 10 to 16 kHz (grouped in 0.5 kHz bins). All curves look very similar. The pre‑treatment and vincristine groups intercept the 6 dB SNR baseline^30^ in the range of f_2_ 15 kHz, the cisplatin group in the range of 13.5 kHz, and the carboplatin group in the range of 14 kHz. Noticeably, in the cisplatin group, the OAEs in the lower frequency range (f_2_ 2.5 and 3 kHz) increase significantly compared with those of the pre‑treatment group, as determined by the Kruskal–Wallis test with Bonferroni-corrected pairwise comparisons, and are significantly reduced in the upper frequency range (from 13 kHz). In the carboplatin group, significantly lower OAEs at f_2_ 4 kHz were registered after the first treatment. In the vincristine group significantly higher OAEs were observed only at f_2_ 3.5 kHz, as determined by Kruskal–Wallis testing with Bonferroni correction.

Averaged SNR (± SE) levels of DPOAEs from 2 to 8 kHz and from 10 to 16 kHz (in 0.5 kHz bins) were calculated for each subject across the three age groups and are presented in Table 3. Across all age groups, pre‑treatment SNRs showed comparable values in both frequency ranges. Following the first treatment with cisplatin, children aged 2–5 years exhibited increased SNRs in the 1–8 kHz range, whereas SNRs in the 10 to 16 kHz range decreased across all age groups. After the second cisplatin cycle, SNRs remained reduced in the high‑frequency range, particularly in the oldest group. Carboplatin treatment showed lower SNRs in both frequency ranges after the first and second cycles, most prominently in the youngest group. By contrast, vincristine treatment resulted in higher SNRs in the 1–8 kHz range in all age groups, with consistently elevated values in the high‑frequency range as well. Statistical analysis using ANOVA with Brown-Forsythe correction revealed significant group differences only for the 1–8 kHz range in the 2–5 year age group (*p* ≤ 0.003), while no significant differences were observed in the remaining comparisons.

**Table 3:** Grouped results are expressed as mean with parentheses specifying the number of observed cases in relation to the total number of eligible patients (first value = measured patients; second value = eligible patients) and standard error.

group	Children 2 to 5 years (21f./17 m)				Children 6 to 10 years (12f./31 m)				Children up to 11 years (36f./36 m)			
	1 to 8 kHz [dB]		10 to 16 kHz [dB]		1 to 8 kHz [dB]		10 to 16 kHz [dB]		1 to 8 kHz [dB]		10 to 16 kHz [dB]	
	Mean	SE	mean	SE	Mean	SE	Mean	SE	Mean	SE	Mean	SE
Pre-treatment (22f./20 m)	15.4 (12/12)	0.8	9.8 (12/12)	1.2	14.5 (6/6)	1.7	11.7 (6/6)	1.1	15.0 (24/24)	0.7	10.8 (24/24)	0.7
Cisplatin first (12f./9 m)	19.2 (3/3)	1.1	9.0 (3/3)	1.4	14.6 (7/7)	1.4	5.7 (7/7)	2.2	15.3 (11/11)	1.8	7.4 (11/11)	1.6
Cisplatin second (12f./12 m)	15.1 (4/4)	2.1	6.7 (4/4)	2.7	16.2 (10/10)	1.6	7.1 (10/10)	1.7	13.2 (10/10)	1.9	6.7 (10/10)	1.7
Carboplatin first (3f./10 m)	10.9 (4/4)	1.4	6.0 (4/4)	2.0	16.8 (3/3)	0.8	10.4 (3/3)	1.4	15.3 (6/6)	1.6	8.9 (6/6)	1.8
Carboplatin second (4f./6 m)	9.7 (3/3)	0.8	6.2 (3/3)	2.7	14.1 (4/4)	1.4	9.8 (4/4)	2.3	17.2 (3/3)	0.7	12.3 (3/3)	0.8
VCR first (11f./18 m)	17.7 (7/7)	1.4	12.7 (7/7)	1.9	16.4 (8/8)	1.4	10.8 (8/8)	1.7	14.6 (14/14)	0.9	9.3 (14/14)	0.9
VCR second (5f./9 m)	18.0 (5/5)	2.1	12.6 (5/5)	1.7	15.3 (5/5)	0.9	10.2 (5/5)	2.4	14.4 (4/4)	0.5	9.7 (4/4)	2.0
*p* value (^c^ Brown-Forsythe correction)	0.003*		0.09		0.888		0,189		0.793^c^		0.109	

All calculated levels are expressed in dB. Significant *p*-values are presented in bold with an asterisks, and ^c^ indicates values corrected using the Brown-Forsythe procedure.

The two-way ANOVA (age group × treatment) showed no significant differences after Bonferroni correction. No multivariate analysis of variance (MANOVA) including the two DPOAE measures (2–8 kHz and 10–16 kHz) across the treatment and groups was conducted. The sample sizes within several medication groups were too small to meet the assumptions required for a reliable MANOVA.

In contrast to the age-group-stratified analyses shown previously, the table below displays the pooled DPOAE levels results without age-group differentiation. It provides mean and median thresholds for 2 to 8 kHz and 10 to 16 kHz, together with standard deviations, confidence intervals, and Brown-Forsythe-adjusted *p*-values for the respective treatment conditions. Pre‑treatment measurements showed mean SNRs of 14.6 dB (2–8 kHz) and 10.7 dB (10–16 kHz). After the first cisplatin administration, SNRs increased slightly in the lower frequency range (mean 15.3 dB) but decreased in the higher range (mean 8.7 dB), a pattern that persisted after the second cisplatin cycle (15.1 dB and 7.2 dB, respectively). Carboplatin treatment resulted in lower mean SNRs in both frequency ranges after the first and second cycles, with values around 13.7–13.9 dB (2 to 8 kHz) and 8.0–9.4 dB (10 to 16 kHz). By contrast, vincristine treatment showed higher SNRs, particularly after the second administration, reaching 16.2 dB at the lower frequency and 10.9 dB at the higher frequency. Overall, cisplatin was associated with the most pronounced reduction in high‑frequency SNRs, whereas vincristine showed the highest SNR levels across both frequency ranges. A significant difference between treatment conditions was observed for DPOAE levels 10 to 16 kHz (*p* ≤ 0.01), whereas no significant differences were found for DPOAE levels 2 to 8 kHz (*p* ≤ 0.743).

For the 10–16 kHz DPOAE, the comparison with the pre-treatment group showed a marginally non-significant difference after the first cisplatin treatment (*p* ≤ 0.056), whereas a significant reduction was observed after the second treatment (*p* ≤ 0.02; Bonferroni-corrected). This inverse frequency-dependent pattern, already apparent in Fig. 2, is likewise reproduced in the grouped averages presented in Table 4.

**Table 4:** DPOAE SNR with mean, median, standard deviation and 95% confidence interval levels from 2 to 8 kHz (left side) and 10 kHz to 16 kHz (right side), across 0.5 kHz bins, are listed in the columns.

	**DPOAE [dB] 2 to 8 kHz**					**DPOAE [dB] 10 to 16 kHz**				
				**Confidence interval**					**Confidence interval**	
	**Mean**	**Median**	**SD**	**Lower**	**Upper**	**Mean**	**Median**	**SD**	**Lower**	**Upper**
Pre-treatment	14.6	14.4	3.6	13.4	15.9	10.7	11.2	3.7	9.4	12.0
Cisplatin first	15.3	15.4	4.9	13.0	17.5	8.7	8.5	6.4	5.5	11.5
Cisplatin second	15.1	16.1	5.5	12.6	17.6	7.2	6.5	5.2	4.8	9.6
Carboplatin first	13.7	14.3	3.8	11.2	16.3	8.0	9.1	4.3	5.1	10.9
Carboplatin second	13.9	15.4	3.5	11.2	16.3	9.4	11.4	4.3	6.4	12.5
VCR first	15.4	14.6	4.1	13.6	17.2	10.4	11.4	4.6	8.4	12.4
VCR second	16.2	15.4	3.5	14.0	18.5	10.9	10.3	4.7	7.9	13.9
*p* value (^c^ Brown-Forsythe correction)	0.743					**0.001**				

The rows represent the therapy status and all calculated values are expressed in dB. Significant *p*-values are presented in bold with an asterisks, and ^c^ indicates values corrected using the Brown-Forsythe procedure.

A MANOVA did not reveal any significant multivariate effects of therapy or gender on the combined DPOAE measures. The univariate effects of therapy on high‑frequency DPOAE levels are described in the ANOVA section (see Table 4). In addition, no significant main effect of gender was observed for either DPOAE level.

### Distortions product otoacoustic emissions vs. hearing testing and thresholds

The SIOP classification (grades 0–3) describes frequency-dependent hearing loss greater than 20 dB HL. Using the DPOAE method presented here, this classification can be translated into an objective measure. Detectable emissions only up to 3 kHz correspond to grade 3, emissions up to 5.5 kHz to grade 2, emissions up to 7.5 kHz to grade 1, and emissions detectable at 8 kHz or higher to grade 0. Based on this definition, Table 5 compares PTA results with the DPOAE‑based classification.

**Table 5:** shows a fourfold table with classification into no hearing loss/hearing loss by PTA, and a comparison of DPOAE measurements.

		PTA (n = 140)	
		Child has no hearing loss	Child has hearing loss
DPOAE	Child has no hearing loss	116/82.9%	19/13.6%
	Child has hearing loss	2/1.4%	3/2.1%

PTA and DPOAE classifications matched 82.9% of cases. PTA showed a high sensitivity (98%) but a low specificity (14%) due to a substantial proportion of false‑positive findings.

### Correlation between hearing loss / DPOAE and cumulative dose of treatment

A significant two‑sided correlation (*p* ≤ 0.001) emerged between the cumulative cisplatin dose and both PTA (10–16 kHz; Spearman’s ρ ≤ 0.327, positive) and DPOAE (10–16 kHz; Spearman’s ρ ≤ -0.41, negative). No other variables showed significant correlations with any of the treatment measures.

## Discussion

Primary prevention and early detection of hearing loss are essential for supporting long‑term outcomes, including survival, education, and social participation, in children undergoing chemotherapy. In this study, we compared the feasibility of subjective hearing tests in children aged two years and older with an objective method capable of assessing auditory function up to 16 kHz. None of the patients received otoprotective medication (e.g. sodium thiosulfate), ensuring that observed auditory changes reflect the effects of chemotherapy alone^31^.

### Hearing thresholds and SIOP classification

Our findings highlight a fundamental limitation of pure-tone audiometry in very young children: reliable behavioral thresholds are often not achievable, particularly in the age group between two and five years. This challenge is well documented in the literature^32^ and likely reflects both developmental factors and the emotional stress experienced by children and parents during oncologic treatment. Tahmasebi et al. demonstrated this psychological burden before therapy can significantly affect children’s performance in clinical assessments^33^, which may contribute to inconsistent PTA results.

Although the SIOP classification provides a standardized framework for grading ototoxicity^2^, its reliance on behavioral thresholds can lead to misclassification when responses are unreliable. In our cohort, several children met SIOP criteria for mild hearing loss despite lacking objective confirmation. Such discrepancies illustrate how subjective testing alone may prompt unnecessary treatment modifications, including premature cessation of cisplatin.

### Distortion product otoacoustic emissions (DPOAE) up to 16 kHz

DPOAE measurements proved highly feasible across all age groups because they require minimal cooperation. In children receiving cisplatin, we observed a characteristic pattern of reduced high‑frequency emissions accompanied by transiently increased low‑frequency amplitudes. Cisplatin is known to induce outer hair cell degeneration beginning at the cochlear base^30^, but the simultaneous enhancement of low‑frequency emissions represents a novel finding in humans. A similar phenomenon was described in mice by Fell et al.^34^, who reported elevated DPOAE levels in the presence of impaired inner hair cell function. This suggests that activation of the medial olivocochlear efferent system may modulate outer hair cell responses during early ototoxic stress.

High‑frequency DPOAEs above 10 kHz were particularly sensitive to early cochlear changes. Comparable tendencies were observed in children treated with carboplatin, although the effects were less pronounced. These findings support the use of extended‑frequency DPOAEs as an early, objective marker of platinum‑induced ototoxicity. In addition, our results argue for changing the platinum compound to carboplatin in children with early signs of ototoxicity when tumor response and long-term survival will not be affected. This is particularly the case for children with metastatic disease, who are not eligible for sodium thiosulfate antidoting^35^.

### Distortions product otoacoustic emissions vs. hearing testing and thresholds

When comparing DPOAEs with PTA, objective measurements demonstrated substantially greater robustness and consistency. Behavioral testing frequently produced false‑positive indications of hearing loss, especially in younger children, whereas DPOAEs provided a more reliable reflection of cochlear status. This discrepancy explains the limited specificity of PTA in this age group and aligns with previous work by Reuter et al.^36^, who also reported inconsistencies between behavioral and objective measures in pediatric cisplatin monitoring.

Importantly, we did not observe objective ototoxicity in children receiving vincristine, consistent with the notion that reported cases of hearing loss during vincristine therapy^3,5,37^ may arise from mechanisms unrelated to platinum‑induced cochlear injury^38^.

Overall, the stability of DPOAE measurements across age groups and treatment protocols underscores their clinical value. Compared with PTA, DPOAEs showed markedly lower variability, reinforcing their suitability for routine monitoring in pediatric oncology.

### Clinical implications

Taken together, our findings demonstrate that objective high‑frequency DPOAE measurements provide early and reliable indicators of ototoxicity, even when behavioral thresholds remain inconclusive. This early detection window is clinically relevant because it may support timely treatment adjustments or the consideration of otoprotective strategies such as sodium thiosulfate^39^ or a change in the chemotherapy regimen to drugs with lower ototoxicity potential. Integrating objective monitoring into routine clinical practice may therefore help prevent long‑term auditory morbidity and support better quality of life for affected children.

The American Speech-Language-Hearing Association and the American Academy of Audiology guidelines recommend the use of both behavioral and objective measures of auditory function as part of the baseline evaluation, since some patients may not be able to provide reliable behavioral thresholds during treatment^40^. Indeed, it was previously reported that many patients (up to 30%) receiving chemotherapy may not be able to adequately participate in ototoxicity screening or respond appropriately during testing because of ongoing disease/treatment effects^32^. Although the oncologist might have the option to adjust the chemotherapy to a potentially less ototoxic regimen^7^ or use otoprotective medications, an unnecessary therapeutic change based on the high drop-out rate in the PTA may influence the outcome of the underlying cancer.

### Limitations

Our findings derive from a single-center study, reflecting the fact that the specific method for measuring DPOAEs was developed by one scientific working group^27^. This may reduce the generalizability of our results. On the other hand, this method can be easily disseminated to audiologists who care for pediatric cancer patients.

We excluded patients under 25 months of age for practical reasons, such as the difficulty of performing PTA in young children. Based on our clinical experience, DPOAEs can be reliably measured in sedated infants and toddlers if obtaining these results is necessary.

## Conclusion

Taken together, these findings highlight that the central objective is to establish protective strategies capable of preventing platinum‑induced ototoxicity while preserving the full antitumor activity of these agents^9^.

This study confirmed that ultra-high-frequency audiograms up to 16 kHz are very suitable for detecting early ototoxic reactions. Unfortunately, this method can only be used to a very limited extent, especially in young children undergoing chemotherapy, because of its high drop-out rate. By contrast, DPOAE measurement can be recommended as a reliable and feasible method for monitoring ototoxicity and detecting it as early as possible, particularly in patients receiving cisplatin‑based regimens.

Our findings reliably provide early indicators to avoid long-term toxicity via treatment adaptation or the initiation of preventive measures. We are convinced that greater knowledge and awareness of ototoxicity as a late effect, particularly in cisplatin treatment, will lead to earlier detection and ultimately help maintain patients’ quality of life.

## Author contributions: 

Conception and design: Dietmar J Hecker, Arne Simon, Marc KH Remke. Provision of study materials and patients: Dorothée Krieter, Nadine Oberkircher, Bernhard Schick, Alessandro Bozzato, Arne Simon, Dominik Schöndorf, Norbert Graf, Rhoikos Furtwängler. Data analysis and interpretation: Dietmar J Hecker, Yeliz Akrasu, Dorothée Krieter, Norbert Graf, Rhoikos Furtwängler, Arne Simon. Manuscript writing: Dietmar J Hecker, Arne Simon. Final approval of manuscript: All authors. Accountable for all aspects of the work: All authors

## Data Availability

The datasets used and/or analysed during the current study available from the corresponding author upon reasonable request.
